# ADAR1: from basic mechanisms to inhibitors

**DOI:** 10.1016/j.tcb.2024.06.006

**Published:** 2025-01

**Authors:** Jan Rehwinkel, Parinaz Mehdipour

**Affiliations:** 1 Medical Research Council Translational Immune Discovery Unit, Medical Research Council Weatherall Institute of Molecular Medicine, Radcliffe Department of Medicine, University of Oxford, Oxford OX3 9DS, UK; 2 Ludwig Institute for Cancer Research, Nuffield Department of Medicine, University of Oxford, Old Road Campus Research Building, Roosevelt Drive, Headington, Oxford OX3 7DQ, UK

**Keywords:** ADAR1, RNA editing, innate immunity, MDA5, ZBP1, type I interferon

## Abstract

Adenosine deaminase acting on RNA 1 (ADAR1) converts adenosine to inosine in double-stranded RNA (dsRNA) molecules, a process known as A-to-I editing. ADAR1 deficiency in humans and mice results in profound inflammatory diseases characterised by the spontaneous induction of innate immunity. In cells lacking ADAR1, unedited RNAs activate RNA sensors. These include melanoma differentiation-associated gene 5 (MDA5) that induces the expression of cytokines, particularly type I interferons (IFNs), protein kinase R (PKR), oligoadenylate synthase (OAS), and Z-DNA/RNA binding protein 1 (ZBP1). Immunogenic RNAs ‘defused’ by ADAR1 may include transcripts from repetitive elements and other long duplex RNAs. Here, we review these recent fundamental discoveries and discuss implications for human diseases. Some tumours depend on ADAR1 to escape immune surveillance, opening the possibility of unleashing anticancer therapies with ADAR1 inhibitors.

## Highlights

Adenosine deaminase acting on RNA 1 (ADAR1) converts adenosine to inosine in double-stranded RNA (dsRNA). This process, known as A-to-I editing, occurs at millions of sites.
ADAR1 deficiency causes activation of multiple innate immune dsRNA sensors including melanoma differentiation-associated gene 5, protein kinase R, oligoadenylate synthases, and Z-DNA/RNA binding protein 1. These proteins trigger interferon (IFN) production, cell death, and other effector mechanisms.
Both RNA editing and RNA binding by ADAR1 ‘defuses’ immunogenic dsRNAs, but the relative importance of these mechanisms for specific dsRNA sensors remains to be fully characterised.
The majority of editing sites in humans are in noncoding stem loop structures derived from the transcription of inverted-repeat *Alu* (IR-*Alu*) elements. Whether all or specific IR-*Alu*s and/or other dsRNA such as *cis*-natural antisense transcripts activate dsRNA sensors in cells lacking ADAR1 remains open.
ADAR1 is a promising target for cancer therapy. ADAR1 loss synergises with the antitumour effects of immune checkpoint blockade and epigenetic therapies.

## ADAR1p150 deficiency causes detrimental autoinflammation

**Figure 1:**
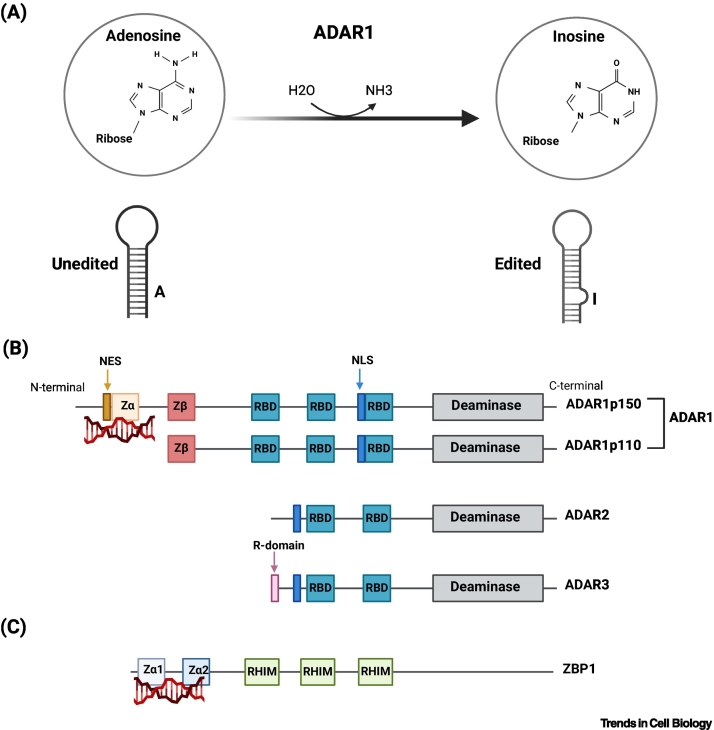
A-to-I editing and domain structures of human adenosine deaminases acting on RNA (ADARs) and Z-DNA/RNA binding protein 1 (ZBP1). (A) ADAR enzymes convert adenosine to inosine via their deaminase domains. (B) All three ADAR family members (ADAR1, ADAR2, and ADAR3) contain a deaminase domain. ADAR1 has three RNA binding domains (RBDs) and ADAR2 and ADAR3 have two RBDs. The ADAR1 p110 and p150 isoforms both contain a Zβ domain, while only ADAR1p150 contains a Zα domain. This isoform shuttles between the nucleus and the cytoplasm due to a nuclear export signal (NES) in addition to the nuclear localization signal (NLS) present in both ADAR1 isoforms. This ability of ADAR1p150 to shuttle to the cytoplasm is important for double-stranded RNA (dsRNA) sensing, which mainly occurs in the cytoplasm. ADAR3 also contains an arginine-rich domain (R-domain) that mediates the ability of ADAR3 to bind to single-stranded RNA *in vitro* [102]. (C) ZBP1 comprises three RIP homotypic interaction motifs (RHIMs) mediating the interaction of ZBP1 with other RHIM-containing proteins such as RIPK1 and RIPK3. ZBP1 also includes two Zα domains. Created with BioRender.com.

Three ADAR enzymes have been identified in mammals. ADAR1 is encoded by the *ADAR* gene, ADAR2 is encoded by the *ADARB1* gene, and ADAR3 is encoded by *ADARB2*. While ADAR1 is highly expressed in various tissues, ADAR2 predominantly and ADAR3 exclusively are expressed in the brain. All ADAR family members contain a nuclear localisation signal (NLS), dsRNA binding domains (dsRBDs), and a catalytic deaminase domain (Figure 1). The dsRBDs recruit dsRNAs for subsequent deamination of adenosine (A) at the C6 position. The deaminase domain thereby catalyses the formation of the unusual nucleobase inosine (I). It is noteworthy that ADAR3, unlike ADAR1 and ADAR2, has no catalytic activity. It may inhibit A-to-I editing in the brain; however, ADAR3’s biological functions remain elusive [1]. This review focuses on ADAR1 that has two different isoforms: ADAR1p110, which is constitutively expressed and mainly located in the nucleus, and ADAR1p150, an IFN-inducible isoform with an extended N terminus. A nuclear export signal (NES) is present in this region unique to ADAR1p150, which uses its NES and NLS for shuttling between the nucleus and cytoplasm. Additionally, the ADAR1p150 isoform harbours a Zα domain that binds to **Z-RNA** (see Glossary). Interestingly, the only two known mammalian proteins that contain the Zα domain are ADAR1p150 and ZBP1 [2]. Finally, both isoforms of ADAR1 contain a Zβ domain that adopts a fold similar to the Zα domain but cannot bind to Z-DNA/RNA [3].

ADAR1 deficiency results in profound inflammatory phenotypes characterised by spontaneous cytokine production, including type I IFNs. In humans, *ADAR* loss-of-function mutations and a dominant negative mutation cause Aicardi–Goutières syndrome (AGS) [4]. This severe encephalopathy is characterised by chronic inflammation and production of IFNs that are usually induced transiently during viral infections. ADAR1 deficiency has also been linked with bilateral striatal necrosis (BSN) [5] and dyschromatosis symmetrica hereditaria (DSH) [6]. In mice, full-body *Adar* knockout (KO) is embryonically lethal and embryos display strong induction of IFNs before resorption [7,8]. Mice lacking only ADAR1p150 display similar severe autoinflammatory phenotypes [9., 10., 11., 12., 13.]. ADAR1p150 therefore limits the production of IFNs during homeostasis and prevents autoinflammation. Selected *Adar* gene-targeted mice and their phenotypes are summarised in Table 1 (see also [2]).

**Table 1:** Overview of selected *Adar* gene-targeted mouse models^a^

Mouse model		Mutation	Phenotype	Cause	Refs
*Adar1* KO *Adar*^–/–^ null for both isoforms (p110 and p150)		Deletion of *Adar1* gene (both isoforms)	Embryonic lethality (E11.5–E12.5)	Defects in haematopoiesis, retarded development, failed liver development, widespread apoptosis in various tissues	[7,8,61,64]
*Adar1p150* KO *Adarp150*^–/–^		Deletion of *p150* isoform	Embryonic lethality (E11–E12)	Defects in erythropoiesis, and activation of type I IFN response	[9,11]
*Adar1p110* KO *Adarp110*^–/–^		Deletion of *p110* isoform	High mortality rate observed during the early postnatal stages (around 2 weeks after birth), growth retardation, weak suckling or breathing	*Adar1p110* RNA editing-independent functions	[13]
*Adar1*-editing deficient *Adar*^*E861A*/*E861A*^		A point mutation in the deaminase domain (E861A) of the *Adar1* gene	Embryonic lethality (E13.5)	Failure in erythropoiesis, activation of the MDA5 pathway	[42]
*Adar1* double-KO mice	*Adar*^–/–^ × *Mavs*^–/–^	Deletion of *Adar1* and *Mavs* genes	Rescue of embryonic lethality in *Adar1* KO mice, up to 10 days post-birth Developmental defects in kidney, spleen, small intestine, and lymph nodes	Suppression of type I IFN response and inflammatory phenotype observed in *Adar1* KO	[10,41,43]
	*Adar*^–/–^ × *Ifih1*^–/–^	Deletion of *Adar1* and *Ifih1* (MDA5) genes	Rescue of embryonic lethality up to 7–8 days after birth	Suppression of type I IFN response and inflammatory phenotype; PKR activation contributes to the postnatal death of these mice	[10,12,41,43]
	*Adarp150*^–/–^ × *Mavs*^–/–^	Deletion of *Adar1p150* isoform and *Mavs* gene	Rescue of embryonic lethality up to 20 days post-birth (up to weaning), dysregulated intestinal homeostasis, defects in lymph nodes and spleen	Suppression of type I IFN response and inflammatory phenotype	[10]
	*Adarp150*^–/–^ × *Ifih1*^–/–^	Deletion of *Adar1p150* isoform and *Ifih1* gene	Postnatal lethality	Stress-induced PKR activation	[10]
	*Adar*^*E861A*/*E861A*^ × *Ifih1*^–/–^	A point mutation in the deaminase domain of *Adar1* and deletion of *Ifih1*	Rescue of embryonic lethality in *Adar1* editing-deficient mice (normal lifespan)	Suppression of type I IFN response and inflammatory phenotype; mild nonpathogenic innate immune activation signature	[42,98]
	*Adar*^–/–^ × *Eif2ak2*^–/–^	Deletion of *Adar1* and *Eif2ak2* (PKR) genes	Embryonic lethality	Stress-induced apoptosis independent of PKR	[8]
	*Adar*^*E861A*/*E861A*^ × *Eif2ak2*^–/–^	A point mutation in the deaminase domain of *Adar1* and deletion of *Eif2ak2*	Embryonic lethality	Apoptosis independent of PKR	[8,12]
	*Adar*^–/–^ × *Ddx58*^–/–^	Deletion of *Adar1* and *Ddx58* (RIG-I) genes	Embryonic lethality (no rescue)	Activation of type I IFN response	[10]
	*Adar*^–/–^ × *Ifnar1*^–/–^	Deletion of *Adar1* and *Ifnar1* genes	Embryonic lethality (survival extended to E14.5–E15.5)	Defects in liver, hepatocytes, and hematopoietic cells	[43]
	*Adar*^–/–^ × *Stat1*^–/–^	Deletion of *Adar1* and *Stat1* genes	Embryonic lethality (survival extended to E15.5)	Hematopoietic defects [8]	[43]
*Adar1* triple-KO mice	*Adar*^–/–^ × *Ifnar1*^–/–^ × *Ifngr*^–/–^	Deletion of *Adar1*, *Ifnar1*, and *Ifngr* genes	Embryonic lethality (E14.5–E15.5) Developmentally delayed, defects in liver size and cellularity	Defects in haematopoiesis	[99]
	*Adar*^–/–^ × *Ifih1*^–/–^ × *Eif2ak2*^–/–^	Deletion of *Adar1*, *Ifih1*, and *Eif2ak2* genes	Partial rescue of postnatal death of *Adar1*^–/–^ × *Ifih1*^–/–^ mice	Deletion of MDA5 and PKR suppresses the inflammatory responses	[12]
	*Adarp150*^–/–^ × *Ifih1*^–/– ×^*Eif2ak2*^–/–^	Deletion of *Adar1p150* isoform and *Ifih1* and *Eif2ak2* genes	Deletion of PKR rescues the postnatal death of *Adar1p150*^–/–^ × *Ifih1*^–/–^ mice and they survive to adulthood	Deletion of MDA5 and PKR suppresses the inflammatory responses	[12]
	*Adar*^*–/–*^ × *Mavs*^*–/–*^ × *Zbp1*^*Zα1α2*/*Zα1α2*^	*Adar1*^*–/*^ ; *Mavs*^*–/–*^ mice that express ZBP1 containing two amino acid substitutions in the two N-terminal Zα domains [N46A and Y50A in the first (Zα1) domain and N122A and Y126A in the second (Zα2) domain]	Extended survival compared with *Adar1*^–/–^; *Mavs*^–/–^ mice; lower birth weights than wild-type (WT) mice; around half of the mice survive until about 20 weeks of life; however, they remain smaller than their control littermates	Inflammatory responses	[61]
*Adar1p150* Zα domain-mutant mice	*Adar*^*mZα*/*mZα*^	Bearing two point mutations in the Zα domain of *Adar1p150* (N175A or N175D and Y179A)	Viable, fertile, and developmentally normal	Normal haematopoiesis and development, spontaneous induction of type I IFN signalling in 8–10-week-old animals in multiple organs and cell types	[60,61,75]
	*Adar*^*W197A*/*W197A*^	W197A mutation in Zα domain of *Adar1p150*	Growth retardation after birth, abnormal development of multiple organs, AGS-like encephalopathy, impaired haematopoiesis	Reduced RNA editing activity and abolished Z-RNA binding, high expression of ISGs	[76]
	*Adar*^*mZα*/–^	Hemizygous expression of Zα domain mutant compounded with an *Adar1* null allele	Die postnatally Smaller offspring and showed the presence of milk in their stomachs	Lethal MAVS-dependent type I IFN response	[60,61]
	*Adar*^*P195A*/*P195A*^	P195A mutation in Zα domain of *Adar1p150* (corresponds to P193A mutations in humans)	Largely normal and the mutation is well tolerated	In outbred mice [63], MDA5-dependent ISG induction in the brain	[54,62,63]
	*Adar*^*P195A*/–^	The P195A mutation is compounded with an *Adar1* null allele	Die postnatally	Lethal disease mediated by MDA5, LGP2, IFNAR, ZBP1, and PKR	[54]
			Approximately 50% of mice show shortened lifespan and the other 50% are normal	Induction of ISGs; the phenotypes in *Adar*^*P195A*/–^ are rescued with loss of MDA5	[62]
			Growth retardation	MDA5-dependent ISG induction	[63]
	*Adar*^*P195A*/*E861A*^	The P195A mutation is compounded with an *Adar1* editing-deficient allele	Viable and normal weaning weight		[62]
	*Adar*^*P195A*/*p150*−^	A P195A mutation in the Zα domain on one allele of Adar*1p150* and deletion of the other *Adar1p150* allele	Viable up to 40 days postnatal, MDA5-dependent mortality	PKR-dependent activation of ISR	[54]
*Adar1 p150* Zα domain-mutant double-KO mice	*Adar*^*mZα*/–^ × *Mavs*^−/−^	Hemizygous expression of *Adar1* with a mutated Zα domain and null mutation of *Mavs*	Animals appeared healthy, did not show upregulation of ISGs, and reached adulthood without displaying signs of pathology at least up to the age of 15 weeks	Suppression of the severe inflammatory response and associated pathologies	[60,61]
	*Adar*^*mZα*/–^ × *Zbp1*^−/−^ or *Zbp1*^*Zα1Zα2*/*Zα1Zα2*^	*Adar1* as above, combined with ZBP1 KO or Zα domain mutation	Partial rescue of survival and pathology		[61,64]
	*Adar*^*P195A*/*p150*−^ × *Ifih1*^–/–^	A P195A mutation in the Zα domain of one allele of Adar*1p150* and deletion of the other *Adar1p150* allele, combined with deletion of *Ifih1*	Viable		[54]
	*Adar*^*P195A*/*p150*−^ × *Dhx58*^–/–^	*Adar1* as above, combined with deletion of *Dhx58* (encodes LGP2)	Viable		[54]
	*Adar*^*P195A*/*p150*−^ × *Ifnar1*^–/–^	*Adar1* as above, combined with deletion of *Ifnar1*	Viable		[54]
	*Adar*^*P195A*/*p150*−^ × *Eif2ak2*^–/–^	*Adar1* as above, combined with deletion of *Eif2ak2*	Viable		[54]
	*Adar*^*P195A*/*p150*−^ × *Rnasel*^–/–^	*Adar1* as above, combined with deletion of *Rnasel*	RNase L deficiency has no affect mortality or weight loss in *Adar1*^*P195A*/*p150*−^ mice	The contribution of RNase L to the phenotypes in this model may be subtle or cell-type specific	[54]
	*Adar*^*P195A*/*p150*−^ × *Zbp1-a*^–/–^	Cross of *Adar*^*P195A*/*p150null*^ mice to animals lacking ZBP1	Normal fertility and survival but slower gain of weight compared with *Adar*^*P195A*/^*^WT^*^−^ littermates	The absence of ZBP1 prevents the activation of cell death pathways, reducing inflammation and allowing normal development and survival	[65]
*Adar2*^–/–^		Deletion of *Adarb1 gene*	Viable up to 3 weeks postnatal (around 20 days after birth), progressively seizure prone 12 days after birth		[100]
Adar3^exon3^		Deletion of exon 3 of *Adarb2* gene, which encodes the two dsRBDs of ADAR3	Viable and normal with some defects in learning and memory		[101]

a Please also see [2], including for *Adar1* conditionally targeted models.

In the first part of this review, we highlight recent fundamental developments that identified the signalling pathways driving autoinflammation in the absence of ADAR1p150. We further discuss how ADAR1p150 **‘defuses’ dsRNAs** that can activate these pathways, focussing on the endogenous dsRNAs that are substrates of ADAR1 and are immunostimulatory when ADAR1 is disabled. In the second part, we review the role of ADAR1 in disease, focusing on the beneficial and detrimental roles of ADAR1 in cancer. Furthermore, we discuss the exciting prospect of the development of ADAR1 inhibitors, outlining potential strategies to target ADAR1.

## Mechanisms regulating editing by ADAR1

ADAR1-mediated editing occurs in both coding and noncoding RNA substrates, with varying efficiencies across species [14]. ADAR1’s editing activity is modulated by various factors. ADAR1 exhibits a sequence preference based on the flanking sequence of edited As, favouring A or U as the 5′ nearest neighbour (A = U > C > G) [15]. While stable dsRNA regions are crucial for ADAR1 binding and activity, selective editing has been observed in dsRNAs with bulges and mismatches [16]. Recent work suggests that ADAR1 recognises structural disruptions in dsRNAs such as bulges and then introduces A-to-I edits 30–35 nt upstream and downstream [17,18]. This creates additional bulges in long dsRNAs that may serve to propagate editing to additional sites on the same dsRNA.

In addition to the dsRNA substrate, protein–protein interactions impact ADAR1’s activity. ADAR1 forms homodimers via protein–protein interactions involving its third dsRBD, a process that is RNA independent but regulates editing activity [19., 20., 21., 22., 23., 24.]. It is possible that two ADAR1 monomers forming a homodimer bind and edit two RNA substrates independently or the same substrate cooperatively. Another study suggests that multiple ADAR1 molecules simultaneously bind dsRNA in a tandem arrangement and that this determines editing patterns [25]. Moreover, ADAR1 forms heteromeric complexes; for example, with Dicer [26]. Using BioID followed by mass spectrometry, Freund and colleagues determined the interactomes of ADAR1 and ADAR2 [27]. Among many other proteins, this analysis identified a family of DZF-domain-containing factors, including ILF3, ILF2, STRBP, and ZFR. ILF3 interacts with both ADAR1 and ADAR2 in an RNA-dependent manner and acts as a global negative regulator of RNA editing. These findings suggest that numerous proteins regulate ADAR1 activity and editing levels [27]. In summary, ADAR1’s function is linked to its editing preferences, including the structure of its dsRNA substrate, and its ability to dimerise and interact with other proteins. Collectively, these factors are likely to determine RNA editing patterns and ADAR1’s broader impact on cellular physiology and pathology.

## ADAR1p150 deficiency results in the activation of dsRNA sensors

A-to-I editing is an **epitranscriptomic modification** and can be mutagenic if it occurs in coding regions: inosine base pairs with cytosine and is therefore read as guanosine during protein translation. A-to-I edits can thus recode mRNAs and change protein function due to the incorporation of different amino acids. However, most A-to-I editing events occur in not-translated regions of RNA. In particular, transcripts deriving from repetitive elements, such as *Alu* repeat elements in humans, are often edited by ADAR1 [28]. The prevailing model explaining autoinflammation in the absence of ADAR1 is that unedited, cellular dsRNAs accumulate and become available to activate dsRNA sensors of the innate immune system [2,14]. These dsRNA sensors include MDA5, PKR, OAS, and ZBP1 and are discussed individually later. It is noteworthy that, besides adenosine deamination, other naturally occurring RNA modifications, such as cytidine deamination (C-to-U), pseudouridylation (Ψ), and RNA methylations, such as *N*6-methyladenosine (m^6^A), *N*1-methyladenosine (m^1^A), 5-methylcytosine (m^5^C), and 7-methylguanosine (m^7^G), may act as hallmarks of normal cellular RNA. These RNA modifications can reversibly or irreversibly alter base pairing, RNA binding, RNA–protein interactions, and RNA stability [29] and they can prevent the activation of innate immune RNA sensors [30]. For instance, the loss of m^6^A has been shown to induce the formation of long endogenous dsRNA, which activates innate immune responses in haematopoiesis [31]. Additionally, single-stranded RNA (ssRNA) with polyuridine induces innate immune responses via TLR7, an ssRNA-specific endosomal receptor [32]. Incorporation of pseudouridine or *N*1-methylpseudouridine in ssRNA instead of uridine reduces these responses [30,33], further indicating that RNA modifications play a vital role in discriminating self from non-self RNAs.

As is the case for ADAR1, changes in the activities of other RNA modifiers, caused by alterations in their expression or mutations, are implicated in human disease. For instance, changes in m^6^A modification levels are associated with acute myeloid leukaemia initiation and maintenance [34], and altered expression of modifiers, which mediate the m^5^C modification, may promote glioma initiation and progression [35].

### MDA5 induces IFNs in ADAR1 deficiency

RIG-I-like receptors (RLRs) are RNA helicases that bind immunostimulatory RNAs and then signal for IFN induction via the adaptor protein MAVS [36]. Such immunostimulatory RNAs include pathogen-derived RNAs – for example, viral RNAs – and unusual cellular RNAs. RLRs discriminate these from normal cellular RNAs based on their length, structure, and modifications. MDA5 is an RLR that surveys the structure of dsRNAs and identifies immunostimulatory RNAs by mechanisms including dsRNA length. In cell-free settings, recombinant MDA5 protein binds to and forms stable filaments on dsRNAs [37,38]. This assembly is regulated by ATP-dependent dynamics and dsRNA length. MDA5 cannot stably associate with dsRNAs containing bulges or other imperfections. Conversely, dsRNAs with perfect base pairing allow stable MDA5 binding and, at least in the test tube, the formation of MDA5 filaments. This has been suggested to allow engagement of MAVS and downstream signalling [39].

**Figure 2:**
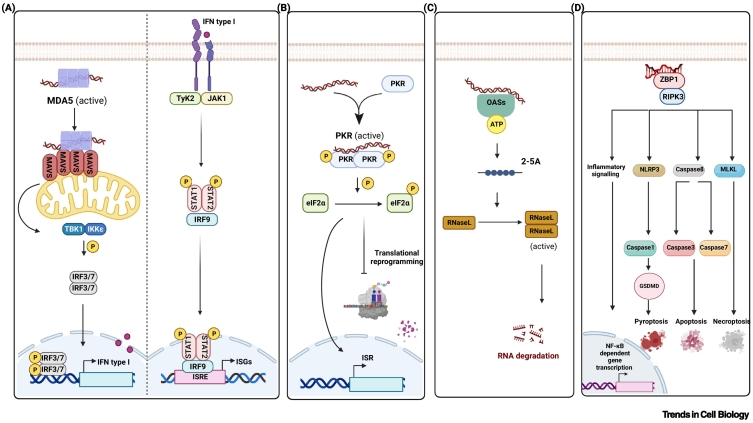
In the absence of adenosine deaminase acting on RNA 1 (ADAR1) or its editing activity, double-stranded RNA (dsRNA) activates various dsRNA sensors. (A) Melanoma differentiation-associated gene 5 (MDA5) forms filaments along unedited dsRNAs, resulting in aggregation and activation of the adaptor protein MAVS. This triggers the activation of TBK1 and IKKɛ kinases, which phosphorylate IRF3 and IRF7. These two proteins, as homo- or heterodimers, translocate to the nucleus, where they induce the expression of type I interferons (IFNs). In an autocrine and paracrine manner, type I IFNs induce the JAK/STAT pathway. The phosphorylated STAT1/STAT2/IRF9 complex binds to IFN-stimulated response elements (ISREs) and thereby induces the expression of IFN-stimulated genes (ISGs) that encode proteins with activities ranging from virus restriction to apoptosis and include protein kinase R (PKR), oligoadenylate synthases (OASs), and Z-DNA/RNA binding protein 1 (ZBP1). (B) Once induced through activation of the IFN pathway, PKR recognises unedited dsRNAs in ADAR1-deficient cells. PKR dimerisation and autophosphorylation then result in its activation. Activated PKR phosphorylates eIF2α, causing translational reprogramming and induction of the integrated stress response (ISR). (C) OAS activation via sensing of unedited dsRNA results in the synthesis of 2′–5′ oligoadenylates (2′-5′As). 2′-5′As activate RNase L that dimerises and degrades RNA, leading to cell death. (D) In the absence of ADAR1, Z-RNA is recognized by ZBP1, resulting in regulated cell death and inflammatory signalling. Created with BioRender.com.

Gain-of-function mutations in the *IFIH1* gene that encodes MDA5 result in its constitutive activation and – much like *ADAR* mutations – cause autoinflammatory diseases, including AGS [40]. Vice versa, the embryonic lethality of mice lacking ADAR1 entirely or ADAR1p150 specifically is rescued at least partially by concomitant KO of MDA5 [10,12,41]. Similarly, ADAR1 editing-deficient (*Adar*^*E861A/E861A*^) mice die during embryonic development and are rescued by additional MDA5 deficiency [42]. MDA5’s key role in mediating disease phenotypes in ADAR1-deficient contexts is further underscored by the requirements for MAVS and MDA5’s cofactor LGP2 [10,41,43,44]. Taken together, these observations suggest that ADAR1’s editing activity safeguards MDA5 from misrecognising cellular RNAs. In this model, endogenous dsRNAs edited by ADAR1 evade MDA5 detection. In the absence of ADAR1 editing, cellular RNAs are detected as immunogenic by MDA5 that then activate MAVS and detrimental IFN production (Figure 2A).

Besides MDA5, additional dsRNA sensors are activated in ADAR1-deficient or -mutated cells and mice, providing insights into the mechanisms underlying ADAR1-associated immunopathology. This includes PKR, OASs, and ZBP1. These and other dsRNA sensors have been extensively reviewed elsewhere [45]. Engagement of these sensors is likely to involve initial IFN production via MDA5 and subsequent induction of PKR, OASs, and ZBP1, all encoded by IFN-stimulated genes (ISGs).

### ADAR1 limits the activation of PKR

The *EIF2AK2* gene encodes PKR, which becomes activated in response to dsRNA binding through dimerisation and autophosphorylation [45]. This allows subsequent phosphorylation of eIF2α, a translation initiation factor, and this inhibits protein synthesis (Figure 2B). Activation of this pathway results in the induction of the **integrated stress response (ISR)**. While inhibiting new protein synthesis generally, the ISR also allows the translation of certain mRNAs, leading to translational reprogramming [46].

ADAR1 negatively regulates PKR activation [47., 48., 49., 50.]. In cancer cells, ADAR1 deficiency can lead to growth arrest and cell death, triggered by activation of PKR, particularly in settings of high baseline ISG expression [51,52]. An interesting question is whether ADAR1’s RNA editing activity prevents PKR activation or whether RNA binding by ADAR1 is sufficient to block PKR. In HEK293T cells, reconstitution experiments with different ADAR1 mutants indicate that both RNA binding and RNA editing are required to fully suppress PKR activation [50]. Similarly, in ADAR1-deficient cancer cells, PKR activation and cell death are only partially reverted by the expression of an editing mutant, ADAR1 E912A (corresponding to E861A in mice) [51,52]. By contrast, a recent study showed in multiple *in vitro* and *in vivo* settings that these ADAR1 editing mutants remain fully competent at blocking PKR activation, suggesting that ADAR1 limits PKR activation in an editing-independent manner [12]. These differences may relate to the repertoires and/or to the levels of endogenous dsRNAs accumulating in different types of cells or in different settings such as IFN stimulation; for example, some ISGs contain dsRNA structures in their 3′UTRs [53].

Taken together these findings highlight an important role of ADAR1 in protecting cells from translational reprogramming induced by endogenous dsRNAs and emphasise ADAR1’s significance in maintaining normal cellular functions. Pharmacological inhibition of the ISR rescued the lethality of *Adar1*-mutated mice (*Adar*^*P195A*/*p150*−^, see below), indicating that ADAR1 prevents detrimental activation of the ISR during homeostasis [54].

### The OAS–RNase L pathway may be a downstream effector of ADAR1 loss

Another family of dsRNA binding proteins are the OASs. These IFN inducible enzymes include OAS1, OAS2, and OAS3. Binding of dsRNA activates the enzymatic activity of OASs and this results in the synthesis of 2′−5′ oligoadenylate (2′-5′A), a small signalling molecule [55]. 2′-5′A then activates the endonuclease RNase L that degrades RNAs, leading to cell death [45] (Figure 2C). In a lung cancer cell line, lethality triggered by KO of ADAR1 is rescued by ablation of RNase L [56]. Additionally, the OAS/RNase L pathway mediates the immune-related cytotoxicity triggered by DNA methyltransferase inhibitors (DNMTis) such as 5-azacytidine (AZA). Demethylation induced by DNMTis derepresses silenced repeat elements, leading to dsRNA formation and OAS/RNase L-mediated cell death. Depletion of ADAR1 enhances this effect [57]. In line with this observation, pharmacological targeting of RNase L rescues cell viability in the absence of functional ADAR1 [58]. However, in contrast to these studies in human cancer cell lines, KO of RNase L does not prevent the growth defects and mortality of *Adar*^*P195A*/*p150*−^ mice [54]. Future studies should delineate in which contexts the OAS–RNase L pathway contributes to pathology on ADAR1 loss and whether editing and/or RNA binding by ADAR1 mediates such effects.

### ZBP1 activates cell death and inflammation in the absence of ADAR1

An exciting recent development is the finding that ZBP1 activation is limited by ADAR1. Like MDA5, PKR, and OASs, ZBP1 is a sensor for dsRNA. However, while the former all detect dsRNA in the conventional A conformation, ZBP1 recognises Z-RNA via its two N-terminal Zα domains. ZBP1 then induces both regulated cell death pathways and an inflammatory gene expression programme (Figure 2D). We refer the reader to recent in-depth reviews on ZBP1 and on cross-regulation of ADAR1 and ZBP1 [2,59] and summarise only the key findings here.

In some patients with AGS, one *ADAR1* allele harbours the P193A mutation in the Zα domain (P195 in mice) and the other allele has a mutation in the deaminase domain. This compound heterozygous state can be mimicked in mouse models. Crossing strains mutated in the Zα domain with *Adar1*^−/−^ or *Adar1p150*^−/−^ mice results in early postnatal lethality accompanied by severe inflammation, albeit with incomplete penetrance in one line of inbred *Adar*^*P195A*/*p150*−^ mice and not in outbred mice [54,60., 61., 62., 63.]. Additional KO of ZBP1 or mutation of its Zα domains largely rescues survival [61,64,65]. Mechanistically, ADAR1-deficient cells accumulate Z-RNA in an IFNAR-dependent manner [53]. Z-RNA then activates ZBP1, and it is likely that both A-to-I RNA editing of dsRNAs prone to adopt the Z conformation and editing-independent functions of ADAR1 explain this effect [53].

## Defining immunogenic dsRNAs edited by ADAR1

Collectively, the data summarised above establish that dsRNA sensors misrecognise endogenous dsRNA in ADAR1-deficient cells and that their downstream effector mechanisms mediate pathology when activated chronically in this setting. Both sequestration of dsRNA and A-to-I RNA editing explain ADAR1’s inhibition of innate immunity, with the relative contributions of these mechanisms likely to depend on context and dsRNA sensor. What are the immunogenic and disease-relevant dsRNAs ‘defused’ by ADAR1 in these ways? The majority of editing events catalysed by ADAR1 in human cells occur in transcripts derived from *Alu* elements and in mice in SINE B1 and B2 repeat elements [16,66., 67., 68.]. The human genome harbours more than 1 million *Alu* repetitive elements that are ~300 nt in length. Many *Alu* elements are found in introns and 3′UTRs and may be present adjacent to each other in an inverted orientation within the same transcript. Such **inverted-repeat *Alu*s (IR-*Alu*s)** form hairpins containing extended stretches of base-pairing and are often edited [14]. Ahmad and colleagues suggested that unedited IR-*Alu*s accumulating in *ADAR1*-mutated cells activate MDA5, explaining IFN responses in diseases such as AGS [69]. When introduced into cells by transfection, unmodified IR-*Alu*s also trigger ZBP1 activation [64].

It is noteworthy, however, that IR-*Alu*s do not form perfect dsRNA structures due to sequence divergence. Mismatches in IR-*Alu*s may prevent or limit MDA5 activation that, at least in biochemical assays, is most potently activated by perfect dsRNA [39]. Moreover, the levels of editing of IR-*Alu*s are often low ([14] and references therein) and ADAR1p150 – the isoform relevant to preventing innate immunity – edits only a subset of all ADAR1 sites [70,71]. It is therefore possible that only some IR-*Alu*s and/or other dsRNAs are immunogenic in the absence of ADAR1 [14,72]. When overlapping genes are transcribed in opposite directions, the resulting ***cis*-natural antisense transcripts (*cis*-NATs)** can form dsRNA. Based on genetic association with diseases, IR-*Alu*s and *cis*-NATs have been proposed to contribute to immunogenic dsRNA formation [72,73]. Additionally, a recent study showed that some cell types, such as human neurons, are intrinsically enriched for immunostimulatory dsRNAs compared with other cell types [74]. This enrichment in neurons is attributed to lengthened 3′UTRs, which may give rise to immunostimulatory dsRNAs as they harbour *Alu* elements or form dsRNA by incorporating complementary sequences from adjacent genes. Such immunostimulatory dsRNAs arising from long 3′UTRs also act as substrates for ADAR1 [74].

Another important feature of immunogenic dsRNAs targeted by ADAR1 may be their propensity to adopt the Z conformation. Mutation of ADAR1’s Zα domain triggers MDA5-dependent IFN responses in animal models, albeit at low levels not causing overt disease [60,75,76]. Moreover, heterozygous Zα domain mutations contribute to severe pathology in humans and mice when combined with KO alleles or alleles that abrogate editing, a phenotype that in mice is MDA5, PKR, and ZBP1 dependent [4,54,60., 61., 62.,^64^,^65^]. These observations suggest that ADAR1-mediated RNA editing causes structural changes in endogenous dsRNAs and that this contributes to protection against immune activation. Interestingly, ADAR1p110 facilitates the resolution of **R-loops** by editing adenosines in RNA: DNA hybrids, with important roles in maintaining telomers in a cancer context [77].

## The role of ADAR1 in cancer

ADAR1 has been described to regulate cancer progression by editing transcripts from oncogenes and tumour suppressor genes, endogenous dsRNAs, and miRNAs. This in turn can either promote or suppress cancer cell proliferation. For instance, ADAR1-mediated editing of the antizyme inhibitor 1 (AZIN1) mRNA results in the incorporation of a different amino acid, increasing AZIN1’s binding affinity to antizyme [78]. This inhibits the tumour suppressive role of antizyme, thereby promoting cancer cell proliferation and survival in various cancer types [78., 79., 80.]. Conversely, recoding by ADAR1p110 of the GABA_A_ receptor α3 (Gabra3) mRNA, which is highly expressed in the adult brain and breast cancer, suppresses invasion by and metastasis of breast cancer cells. The GABRA3 protein encoded by the A-to-I-edited form of its mRNA thus has a tumour suppressive role [81]. A detailed review of mRNAs and miRNAs, which are edited by ADAR1 and impact cancer proliferation and survival, is available [82]. While the loss of ADAR1 or its editing activity indicates tumour sensitivity to ADAR1 depletion in a number of settings, direct evidence for the oncogenic role of ADAR1 through gain-of-function experiments remains elusive and worthy of future studies.

Recently, roles of ADAR1 beyond tumour progression and metastasis have been described. Rivera and colleagues suggested that ADAR1 promotes the tumorigenesis of leukaemic initiating cells (LICs) and contributes to relapse in patients with T cell acute lymphoblastic leukaemia (T-ALL) [83]. In this setting, RNA editing and sequestration of dsRNAs in the nucleus suppress the activation of cytoplasmic dsRNA sensors. These findings suggest that ADAR1 could be a target to improve T-ALL treatment and that ADAR1 inhibition could prevent tumour relapse and therapy resistance [83].

### Tumour cells require ADAR1 to escape antitumour immunity

Cancer cells often show high mutation rates and employ various mechanisms to evade the host’s antitumour immune responses. One such mechanism involves hijacking the factors responsible for maintaining immune system balance and homeostasis. ADAR1 is one of these factors and controls the activation of innate immune responses to pathogenic RNAs or endogenous RNAs that resemble viral RNAs.

Exploring the interplay between IFN signalling in tumours and the vulnerability of cancer cells to dsRNA-induced stress revealed that intrinsic IFN production by cancer cells primes them to depend on ADAR1 for survival. Cancer cell death can occur on loss of ADAR1, mediated through the IFN pathway and PKR. This suggests that inhibiting ADAR1 as a standalone target could represent a novel approach for the treatment of a subset of cancer cells without the need to combine it with other cancer therapies [51,84]. Moreover, targeting ADAR1 could be a potential strategy for combination therapies. For instance, targeting ADAR1 has been shown to enhance the vulnerability of cancer cells to immune checkpoint blockade. Immune checkpoint blockade aims to unleash the adaptive immune system’s ability to identify and eradicate cancer cells. Recent studies have revealed that targeting ADAR1 in cancer cells enhances the efficacy of immune checkpoint blockade therapy by increasing immune cell infiltration. This effect was achieved through the activation of MDA5 and inhibition of tumour cell growth via the activation of PKR [52]. These studies collectively highlight how ADAR1-mediated RNA editing helps cancer cells to escape antitumour immune responses, thereby contributing to tumour progression and resistance to immunotherapies [51,52,84].

Another promising combination therapy involving ADAR1 is the treatment of cancer cells with epigenetic therapies, such as the DNMTi 5-aza-2′-deoxycytidine (also known as 5-AZA-CdR or decitabine). Administering DNMTis to cancer cells can trigger the cryptic transcription of IR-*Alu*s, leading to the formation and accumulation of dsRNAs that ADAR1 can act on [85]. This accumulation of endogenous dsRNAs induces dsRNA stress, prompting the activation of antiviral immune responses against cancer cells. This cell state is known as ‘**viral mimicry**’ and occurs because the self dsRNAs are thought to resemble viral RNAs, thereby stimulating innate and adaptive immune responses against cancer cells [86,87]. Simultaneously, inhibition of ADAR1, a negative regulator of the immune system, synergises with DNMTis [85,88] and potentially other epigenetic therapies to amplify the viral mimicry response.

As mentioned earlier, the viral mimicry response can be activated by the accumulation of endogenous dsRNAs. This activation may occur through the targeting of repressive epigenetic marks that silence retroelements. Additionally, spliceosome-targeted therapies result in the retention of intronic repeat elements in spliced mRNAs, which are then exported to the cytoplasm and form dsRNA. In this way, they stimulate the viral mimicry response [89]. Furthermore, the 5′-to-3′ exoribonuclease XRN1 modulates the abundance of endogenous dsRNAs and thereby regulates the viral mimicry response [90,91]. Taking these findings together, ADAR1 and other factors mediating the viral mimicry response are attractive targets for therapeutic intervention.

## Development of ADAR1 inhibitors

**Figure 3:**
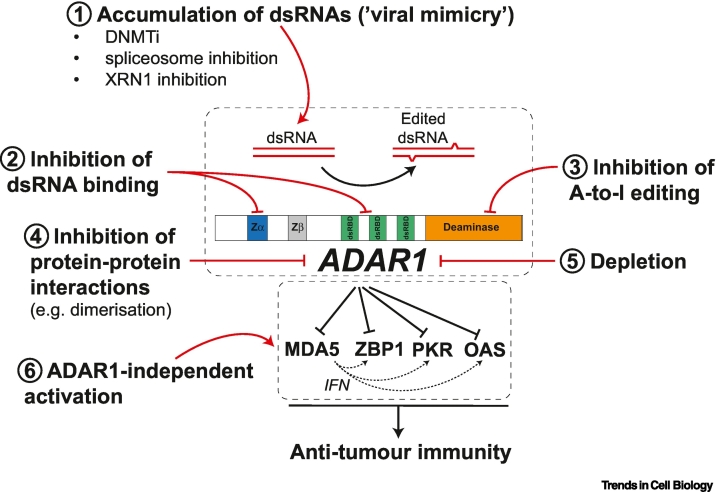
Potential strategies to target adenosine deaminase acting on RNA 1 (ADAR1) for cancer therapy. ADAR1 binds and/or edits endogenous double-stranded RNAs (dsRNAs). This limits antitumour immunity by preventing the engagement of dsRNA sensors including melanoma differentiation-associated gene 5 (MDA5), Z-DNA/RNA binding protein 1 (ZBP1), protein kinase R (PKR), and oligoadenylate synthases (OASs). This protumoral role of ADAR1 could be disabled in at least six different ways. (1) Inhibitors of DNA methyltransferases (DNMTis), of the spliceosome, or of nucleases such as XRN1 elevate levels of endogenous immunogenic dsRNAs. This may saturate ADAR1’s editing capacity, allowing dsRNA sensing. (2–4) Inhibition of dsRNA binding to ADAR1, its deaminase activity, or protein–protein interactions may result in the accumulation of unedited dsRNAs available for sensing. (5) ADAR1 depletion would similarly result in an accumulation of unedited dsRNAs. This could be mediated by ADAR1-targeting PROTACs [103] or perhaps by modulation of ADAR1 pre-mRNA splicing [95]. (6) ADAR1 may be bypassed by downstream activation of dsRNA sensors. For example, ZBP1 can be activated by CBL0137 [53].

Efforts to develop ADAR1 inhibitors (Figure 3) are underway and often focus on inhibiting its deaminase activity. For example, a short RNA duplex containing the nucleoside analogue 8-azanebularine is a potentially selective inhibitor of ADAR1’s deaminase activity [92]. The activity of this compound or its derivatives needs to be systematically assessed in cell-based assays. Additionally, assessing the safety profile of targeting editing mediated by both ADAR1 isoforms in transformed cells compared with normal cells is crucial to determine the compound’s safety window. ADAR1p150 regulates biological processes distinct from ADAR1p110 that controls organ development and homeostasis [10,13] and, as discussed earlier, ADAR1p150 acts not only through RNA editing but also through its RNA binding activity and its cytoplasmic localisation. Targeting the IFN inducible isoform ADAR1p150 would likely reduce off-target effects on organ development/homeostasis potentially associated with targeting both isoforms and might broaden the spectrum of therapeutic indications.

A high-throughput virtual screen identified lithospermic acid and regaloside B as potential inhibitors that interact with the Zα domain of ADAR1. However, systematic functional assays are required to confirm these interactions and to characterise the cell-based phenotypes associated with these compounds [93]. A potential selective inhibitor of the ADAR1p150 isoform, named AVA-ADR-001, has been identified [94]. This inhibitor directly binds to the Zα domain of ADAR1p150 and induces IFN in cells. AVA-ADR-001 has antitumour effects *in vivo* and shows synergistic effects when used in combination with anti-PD1.

In parallel, alternative approaches to direct targeting of ADAR1 may be fruitful. One example is the use of rebecsinib, also known as 17S-FD-895. Rebecsinib is a pre-mRNA splicing modulator and targets the SF3B protein complex, a component of the spliceosome. Analysing haematopoietic progenitor cells (HPCs) from patients with myeloproliferative neoplasms, Crews and colleagues found higher expression of ADAR1p150 at mRNA and protein levels than in normal HPCs [95]. Interestingly, rebecsinib blocks the expression of ADAR1p150 in this setting. Concomitantly, rebecsinib inhibits the self-renewal of leukaemic stem cells, which are thought to require ADAR1p150. Rebecsinib shows minimal or no effect on normal HPCs [95]. Rebecsinib could thus be a promising therapeutic approach for haematological malignancies. However, mechanistic studies will be required to understand the molecular basis of rebecsinib’s effects. Crews and colleagues speculate these might be related to effects on post-transcriptional processing of *ADAR1* mRNA [95], but splicing modulation of other transcripts may be important, too. Moreover, rebecsinib’s efficacy in solid tumours should be assessed. It will also be interesting to test other splicing inhibitors that have been designed based on recurrent splicing factor mutations observed in myelodysplastic syndromes, such as in *SF3B1*, *SRSF2*, *U2AF1*, and *ZRSR2* [96]. Another approach is the use of the curaxin CBL0137, which was identified as a ZBP1 activator [53]. This compound induces Z-DNA formation and consequently activates ZBP1 and ZBP1-mediated necroptosis. As such, CBL0137 mimics ADAR1 inhibition by generating an agonist for a downstream nucleic acid sensor.

## Concluding remarks

Since the identification of the ADAR family in 1987 [97], significant progress has revealed the functions and activities of these proteins, mapped A-to-I RNA editing sites, and uncovered roles in autoimmune diseases and cancer. However, there are many unanswered questions that require future studies (see Outstanding questions). Here, we reviewed recent developments in ADAR1 research, focusing on the signalling pathways driving inflammatory responses on loss of ADAR1p150 and the sources of immunogenic dsRNAs that ADAR1 binds and/or edits and thereby defuses. We also highlighted recent findings on ADAR1’s role in cancer progression, immune evasion, and resistance to therapies. Additionally, we summarised recently identified potential inhibitors of ADAR1. Inhibitors that directly or indirectly affect the activity of ADAR1 hold substantial promise for cancer treatment and may perhaps also be used in viral infections. However, there remains a significant need to develop novel ADAR1 inhibitors, ideally with specificity for ADAR1p150. Targeting the Zα domain or perhaps strategies that target only the cytoplasmic pool of ADAR1 could offer a route to achieve this selectivity.

Outstanding questions Which of the dsRNA substrates of ADAR1 are immunogenic, and why? Future studies should consider topological features, length, abundance, and levels of A-to-I editing. Are these features cell-type dependent or are they generalisable?

What are the relative contributions of MDA5, PKR, OASs, and ZBP1 to pathology in ADAR1 deficiency? What is the contribution of RNA binding and RNA editing by ADAR1 to the repression of these sensors? Do these dsRNA sensors recognise overlapping or distinct dsRNAs and do they compete with ADAR1 for dsRNA binding?

What happens to dsRNAs edited by ADAR1? Is RNA half-life or subcellular localisation affected by A-to-I editing? If yes, what are the underlying mechanisms?

Which ADAR1 domains should be targeted for optimal clinical effects? Is inhibition of ADAR1 deaminase activity tolerable for normal cells or is this strategy that targets both ADAR1p150 and ADAR1p110 cytotoxic? Can the Zα domain or another feature unique to ADAR1p150 such as its cytoplasmic localisation be targeted?

What is the safety window for using ADAR1 inhibitors to kill cancer cells without adverse effects on normal cells? Can ADAR1 inhibitors be used to induce innate immunity to prevent or treat virus infection?

Do RNA modifications other than A-to-I editing regulate the formation of endogenous dsRNAs? Do they suppress innate immune responses under homeostatic conditions, and if so, is their role upstream/downstream of, in parallel with, or independent of ADAR1? What are the endogenous RNAs modified and are there mechanisms of self and non-self discrimination by innate immune receptors?

Alt-text: Outstanding questions

## Glossary

***Cis-natural antisense transcripts (cis*-NATs)**: generated by transcription of genes found in the same genomic location on the + and – strands of the DNA genome. *cis*-NATs are very rare in humans but have the potential to form perfect dsRNAs.
**‘Defusing’ dsRNA**: the process of dsRNA editing by ADAR1 that leads to A-to-I conversion, thereby creating a U:I base pair that changes dsRNA secondary structure. This leads to reduced recognition by dsRNA sensors that mediate antiviral signalling. Therefore, ‘defused’ dsRNAs do not trigger innate immune responses.
**Epitranscriptomic modification**: chemical modifications that occur on RNA post-transcriptionally. The majority of these modifications are reversible, while A-to-I editing is irreversible. RNA modifications can impact RNA structure, stability, localisation, and biochemical interactions and a variety of cellular processes such as splicing and translation.
**Integrated stress response (ISR)**: a conserved signalling pathway in eukaryotes activated by a range of physiological and pathological stimuli, such as viral infection, amino acid or haem deprivation, and endoplasmic reticulum stress. On activation, this pathway induces translational shut down through phosphorylation of eIF2α. While this inhibits the synthesis of most proteins, it simultaneously allows the expression of specific genes that mediate the ISR such as *ATF4*. Overall, activation of the ISR restores cellular homeostasis during stress.
**Inverted-repeat *Alus (*IR-*Alu*s)**: *Alu*s are repeat elements in the human genome that belong to the short-interspersed-nuclear element (SINE) class of repeats. IR-*Alu*s are pairs of *Alu*s present in inverted orientation in adjacent genomic locations. Transcription of IR-*Alu*s results in RNAs that can form stem loop structures containing extended stretches of dsRNA.
**R-loops**: three-stranded nucleic acid structures formed by the hybridisation of an RNA transcript with its template DNA strand, leading to the formation of an RNA–DNA hybrid and displacement of the non-template DNA strand. Formation and resolution of R-loops play an important role in maintaining genomic integrity.
**Viral mimicry**: in the context of cancer cells, a cellular state in which accumulation of dsRNAs derived from endogenous retroelements, resembling viral RNAs, activates innate immune RNA sensors. Consequently, this leads to the activation of antiviral signalling, induction of IFNs, and cancer cell death.
**Z-RNA**: a left-handed double helical conformation of dsRNA with a zigzag-shaped phosphodiester backbone. dsRNA normally forms a right-handed double helix known as A-RNA, which is thermodynamically more stable than Z-RNA. However, sequence composition, salt concentration, and binding to Zα domains can stabilise Z-RNA. Zα domains are present in two human proteins, ADAR1p150 and ZBP1, and several viral proteins.
